# Functional Outcome of Gastrointestinal Tract and Quality of Life After Esophageal Reconstruction of Esophagus Cancer

**DOI:** 10.4103/1319-3767.45050

**Published:** 2009-01

**Authors:** Manochehr Aghajanzadeh, Feizollah Safarpour, M. Reza Koohsari, Farborz M. Ghanaei, Sadigheh M. Bodaghi, Hadi Tozandehgani

**Affiliations:** Department of Thoracic, Guilan University of Medical Sciences, Razi Hospital Rasht, Iran; 1 Department of General Surgery, Guilan University of Medical Sciences, Razi Hospital Rasht, Iran; 2 Guilan University of Medical Sciences, Gastrointestinal and Liver Diseases Research Center, Iran

**Keywords:** Carcinoma of esophagus, esophageal reconstruction, quality of life, transhiatal esophagectomy

## Abstract

**Background/Aim:**

Information about functional outcome and quality of life after esophagectomy and esophageal reconstruction (ER) for the treatment of esophageal cancer, as evaluated by the patients themselves is limited. We aimed to study the post-surgical outcome of such patients to detect for the development of any complications that may arise from the surgery as well as to evaluate their quality of life following the surgery.

**Methods:**

From 1993 to 2003, 240 patients with stage I, II, or III esophageal carcinoma underwent esophagectomy at Razi Teaching Hospital located in the north of Iran. Of these, 192 patients filled out a questionnaire during a 2-year period (ranging from 12 to 48 months after surgical reconstruction). Among them, there were 134 men (69%) and 58 women (31%), and the mean age at the time of ER was 48 years (ranging from 22 to 75 years). Transhiatal esophagectomy, extended esophagectomy (three field operation), and Ivor-Lewis resection were done in 142 (73.95%), 30 (15.62%), and 20 patients (10.42%), respectively. Intestinal continuity after esophageal resection was established with stomach in 154 patients (80%), colon in 28 patients (14%), and small bowel in 10 patients (5.2%). Cervical anastomosis was established in 172 patients (89.6%), while intrathoracic anastomosis was performed in 20 patients (10.4%).

**Results:**

After ER, 66 patients (34.4%) suffered from dysphagia to solids and 50 patients (26%) required at least one or three postoperative dilatations for alleviation of symptoms. Gastroesophageal reflux was seen in 32 patients (16.66%) and was more common in thoracic anastomosis patients than in cervical anastomosis patients. Heartburn was present in 33 cases (17%), 30 of whom required medication (37%). The number of meals per day was three to four in 116 patients (60%), more than four in 51 patients (29%), and less than three in 19 patients (9.82%). The number of bowel movement per day increased in 52 patients (27%), decreased in 60 cases (31%), and unchanged in 80 patients (41%). Weight gain was reported by 38 patients (19.8%), and weight loss was reported by 50 patients (26%). No change in weight occurred in 100 patients (52%). Overall satisfaction was excellent in 29 patients (15%). Overall quality of life (work, pain-relief, vitality, and emotional status) was lower than in general population. Age, sex, and stage of cancer did not affect the functional outcome but affected the quality of life. Also patients who received cervical anastomosis and ER with colon had significantly fewer reflux symptoms. Most of the patients with colon reconstruction gained weight.

**Conclusions:**

Self-assessment of postoperative ER by the patients after esophagectomy for malignant disease demonstrates that undesirable symptoms are frequently present at short- and long-term follow-ups. Short- and long-term functional outcome is affected by the type of reconstruction after esophagectomy. Results of this study suggest that colon graft in ER is significantly advantageous compared with other methods because of the ability of patients to gain weight and avoid developing postoperative reflux.

Esophageal carcinoma is one of the most common malignancies in Iran and is frequently found in the north of Iran, especially in areas around the Caspian Sea (180 cases in 100,000 of the population). The incidence of esophageal carcinoma continues to rise in this part of Iran and requires aggressive and timely surgical and oncological treatments. Overall 5-year survival rate at major treatment centers in Iran remains poor.[1] When esophageal cancer presents symptomatically, the disease has a very poor prognosis, but when it is detected early enough, it can have a favorable prognosis. Balloon cytology screening provides early detection and treatment with excellent long-term survival and acceptable quality of life.[2] Early detection and resection of esophageal carcinoma provides the best chance for a cure. Long-term survival is dependant upon the stage of the cancer at the time of diagnosis.[3,4] In the northern part of Iran (Guilan province), nearly all esophageal surgeries were performed by our team here at Razi Teaching Hospital in the city of Rasht, which is affiliated with Guilan University of Medical Sciences and other private hospitals.

In our previous unpublished experimental studies, we found that after esophagectomy and esophageal reconstruction (ER), majority of patients complained of dysphagia, reflux, regurgitation, abdominal pain, and cramps. However, little was known about the short- and long-term functional status and quality of life after resection and ER[4–6] in survivors. Little information exists about gastrointestinal complications and quality of life after ER for esophageal carcinoma. Majority of patients have dysphagia, reflux, aspiration, dumping emptying, pain, activity, and weight loss. The goal of this study is to monitor patients for a short period of time to detect any complications that might have resulted following the operation as well as evaluation of these patients for their quality of life since the operation.

## PATIENTS AND METHODS

From 1993 to 2003, 240 stage I, II, and III patients with esophageal carcinoma underwent esophagectomy and esophageal reconstruction (ER) at Razi, Aria, and Golsar private hospitals in the city of Rasht located northern part of Iran.

Modified standardized questionnaire [Table 1] is a self-administered health assessment tool that permits group comparison in eight different areas including general health, daily activity, work, emotional problems, social activity, nervousness/depression, pain, and vitality. A total of 192 of the patients (i.e. 80%) completed the questionnaires while in the clinics, at their homes, or by telephone. Follow-up data were obtained from the patients in the two-part survey. Part 1 subjectively evaluated patients for any symptoms pertaining to their digestive functions in those who received esophagectomy. Part 2, used the Medical Outcomes Study 36 - Item Short-Form Health Survey (MOS SF - 36).[5]

**Table 1:** The Questionnaire

1. What kind of foods are you now able to eat since the operation?					
	Anything	Soft diet only	Liquid only			
2. Are you able to take three meals a day?					
	Yes	No			
3. Have you maintain your body weight after the operation?					
	Yes	No			
4. Do you have any symptoms related to eating since the operation?					
	Fullness	Nausea	Regurgitation	Dysphagia	
5. Do you need to take any medications to help you relieve your symptoms?					
	Yes	No			
6. What is your overall satisfaction regarding to the operation?					
	Excellent	Good	Fair	Worse	
7. Are you able to sleep comfortably in your regular routine position(s) in bed?					
	Yes	No			
8. How do you rate your daily activities?					
	Good	Fair
9. Are you suffering from any abdominal or chest pains?					
	Yes	No			
10. Do you have more anxiety now compared to the time before the surgery?					
	Yes	No			
11. How do you describe your mood since the operation?					
	Good	Fair			

The records of these patients were analyzed for age, sex, sign, symptoms at the time of initial diagnosis, type of operation, organ of reconstruction, operative complications, mortality, quality of life, and finally functional outcome. Factors influencing functional outcome and quality of life were obtained through the patient's medical records and the surveys. Also signs and symptoms of aspiration, frequency of daily meals, and presence of dumping syndrome, bowel habits, and weight changes as well as the patient's overall satisfaction of the operation were all evaluated.

## RESULTS

Of the 192 patients (80%) completed questionnaire form, there were 134 men (69.80%) and 58 women (30.2%). The mean age at the time of esophagectomy and ER was 48 years (range from 22 to 75 years).

### Presenting symptoms

Dysphagia was the most common symptom (100 %). Other symptoms are shown in Table 2. Grading of dysphagia is shown in Table 3.

**Table 2:** Preoperative symptoms and signs

Symptoms and sign	Number of patients (%)
Dysphagia	192 (100)
Weight loss	80 (41)
Pain	32 (16)
Regurgitation	39 (19)
Aspiration	22 (11)
Respiratory complications	
(abscess, pneumonia)	42 (21)
Odynophagia	16 (8)
Hoarseness	4 (2)

**Table 3:** Grading of dysphagia

Grade	Number of patients (%)
1	30 (15.5)
2	102 (53.5)
3	40 (20.5)
4	20 (10.5)

Thirty patients (15/62%) had previous chemotherapy or radiation therapies.

Diagnosis and staging were performed by barium swallow, endoscopy, CT scan, and ultrasonography of the chest and abdomen. The operative procedure performed on 142 of the patients (73.95%) was transhiatal esophagectomy. Extended esophagectomy (three field operation) was performed on 30 patients (15.62%) and Ivor-Lewis esophagogastrectomy in 20 patients (10.42%). Intestinal continuity after esophageal resection was reestablished with stomach in 154 patients (80%), colon in 28 patients (14.50%), and small bowel in 10 patients (5.22%). The cervical esophagus anastomosis performed in 172 patients (89.58%) and upper and middle thoracic anastomosis in 20 patients (10.62%). A pyloromyotomy was done in 90 patients (46.87%) and a pyloroplasty in 42 patients (21%).

Intraoperative complication occurred in eight patients: tracheal rupture in two, bleeding in three (due to azygos vein rupture), and spleen rupture in three patients. Trachea was repaired by posterolateral thoracotomy and bleeding controlled by reoperation. Postoperative complications occurred in 78 patients (40%), as shown in Table 4.

**Table 4:** Postoperative complications

Complications	Number of patients (%)
Arrhythmia	24 (30.76)
Pneumothorax	20 (25.64)
Pneumonia	15 (19)
Respiratory failure	14 (17.94)
Wound infection	12 (15)
Empyema	14 (17.94)
Anastomotic leaks (Fistula)	13 (16.66)
Myocardial-infarction	11 (14)
Atelectasia	9 (11.53)
Mediastinitis	4 (5)
Chylothorax	4 (5)
RLN damage	3 (3.84)

Feeding jejunostomy was placed in all patients. All cervical and intrathoracic leaks healed with conservative management.

Postoperative pathology reports included squamous cell carcinoma in 152 patients (79%), adenocarcinoma in 37 patients (20%), adenosquamous in 2 patients (1/5%), and leiomyosarcoma in one patient (0.5%).

Average postoperative hospital stay, after ER, was 22 days (range from 10 to 40days). There were 20 mortalities (10.41%). The time interval between ER and survey ranged from 12 to 48 months. Ninety-eight patients (38 %) were still alive at the time of the last follow-up.

One-, two-, three-, and four-year survival rates were 80%, 61%, 52%, and 38%, respectively. Information about functional outcome was obtained from all 192 patients. Dysphagia to solid was mild in 104 patient (61%), severe in 43 patients (25%), and absent in 25 patient (14%). About 104 patients (61%) had dysphagia to pureed foods (i.e., mashed food), and 30 patient (17%) had dysphagia to liquids. Fifty patients (26%) underwent at least two postoperative dilatations, and 40 patients (20.83%) required four to seven dilatations.

Eleven patients (6%) had odynophagia, 33 patients (19%) had heartburn that required antacids for relief of heart burn, 33 patients (6%) had chronic cough, 11 patients (6%) complained of dysphonia, and three patients (1.74%) had hoarseness. The number of meals per day is shown in Table 5.

**Table 5:** The number of meals per day

Number of meals per day	Number of patients (%)
3–4	104 (60)
More than 4	51 (30)
Less than 3	17 (10)

Seventy-nine patients (46%) experienced symptoms of postprandial dumping, including 22 patients (27.5%) with nausea, 36 patients (46%) with cramps and abdominal pain, and 21 patients (26.5%) with diarrhea. Also, 46 patients (27%) failed to regain preesophagectomy weight; 93 patients (54%) maintained preoperative weight, and 33 patients (19%) gained weight compared with their preoperative weight.

Overall satisfaction was excellent in 26 patients (15%), good in 103 patients (60%), fair in 26 patients (15%), and poor in 17 patients (10%).

Age, sex, and anatomic site of tumor did not influence functional outcome, but ER with colon have better influence on the weight gain and symptoms of esophagitis. Patients with cervical anastomosis had significantly fewer symptoms of reflux when compared with those who received intrathoracic anastomosis. Type or absence of gastric drainage did not significantly affect function outcome.

Information of quality of life as assessed by the modified MOS SF-36 was available for 110 patients (63%). Overall physical function score (daily activity, work, emotional problem, social activity, nervousness/depression, pain, and vitality) were decreased significantly compared with national average. Female patients had better quality of life than male patients. Age, time interval from occurrence of tumor to operation, histology, and location of tumor did not significantly affect the quality of life, but the type of the operation, organ for ER, stage of cancer and adjuvant therapy affected the quality of life. Patients who received transhiatal esophagectomy have better quality of life than those with transthoracic esophagectomy. This is because postoperative chest pain is more common when transthoracic approach is used during the operation. Patients with postoperative adjuvant therapy have relatively poor quality of life.

## DISCUSSION

Esophageal cancer is one of the most common cancers of gastrointestinal tract in the north of Iran (180 in 100 000 of population).[1] Esophagectomy is the treatment of choice for cancer of the esophagus.[6] Success of curative treatment of esophageal cancer has traditionally been measured by survival.[7]

Despite continued refinement of operative techniques of esophagectomy, cumulative quality of life after esophagectomy has remained poor in most patients.[8] Until now, only a few reports have taken a look at the quality of life for the patients who have received esophagectomy due to carcinoma of esophagus.[7] A review of the literature by Gelfand and Finley[9] in 1994 revealed that out of 7569 publications on the subject of esophageal carcinoma, only 44 (0.58%) dealt with studying the quality of life[7] following esophagectomy. Similarly, a better understanding of the functional outcome and quality of life is needed in our area of health care system. Moreover, there are only a few self assessment reports about quality of life and functional outcome of patients after the surgery.[10–12]

Esophageal balloon cytology screening is the best approach for early diagnosis of esophageal carcinoma. Surgical resection in the early stage provides excellent long term survival with acceptable quality of live.[2] With this screening program the 5, 10, 15, 20, and 25-year survival rates were 86.14%, 75.03%, 64.48%, 56.17%, and 49.93%.[2]

Although the primary objective of ER is to restore normal swallowing with minimal morbidity and mortality, only a minority of our patients (20%) were completely symptom free two or more years after the esophageal resection and reconstruction. Potential complications of dysphagia, aspiration, regurgitation, chronic cough, delayed gastric emptying dumping, and diarrhea can all adversely influence short and long term functional outcome.[13–14] Several reports have demonstrated excellent or good functional results, postoperatively, in the majority of patients.[15–16] However, comparing these reports and their data are difficult because different tools have been used to evaluate the quality of life and functional out come.[8]

We are in a region where the esophageal cancer is one of most common cancers of gastrointestinal tract and esophageal surgery is becoming increasingly common. Quality of life and functional outcome is obviously of great importance for these patients after their operation.

Health outcome is better measured by using general health measures and traditional biomedical tools synchronously.[7]

In the present study, we prepared a questionnaire for upper and lower digestive system functions along with a quality of life survey. Our study demonstrated that the majority of patients were symptomatic for two years (median: 2). Upper gastrointestinal symptoms such as reflux, dysphagia, heartburn, and regurgitation were complains made by most of the patients. More than (35%) complained from reflux symptoms and 66% of these patients used antacids and had difficulties in swallowing, of whom 37% required dilation. In the absence of any anatomic causes of dysphagia after cervical esophagogastrectomy, a functional etiology may be explained by hypertensive peristalsis measured with a manometer, resulting from distention of the remaining cervical esophageal remnant.[13]

Thirty two percent of the patients reported some degree of dumping. Moreover, the rate of dumping syndromes was higher in women and young patients. In addition, symptoms of dumping and crampy abdominal pain were also common but only 17% had some degree diarrhea and only 9% used medication. Other researchers have also reported similar results.[17,18] They suggest that these postoperative symptoms are not severe in much of the patients who underwent ER.

One significant finding in our study suggests that the incidence of reflux is significantly reduced when the anastomosis is located in the neck.[7] However, a reduction in the late reflux symptoms needs to be weighed against an increased rate of fistula formation and recurrent nerve injury[19,20] in patients with in patients with cervical anastomosis. Moreover, fistula formation had an adverse impact on the quality of life and social functioning scores, which measure physical functioning, health perception, and the need for postoperative dilation.[6]

Thirty percent (30%) of our patients regained their preoperative weight and 38 patients (19%) had weight gain. All this eventually prompted us to conclude that for post operative weight gain, ER with colon is superior to other methods (i.e. stomach and small intestine). Cough, aspiration, and uncomfortable sleep were seen in 30% of the patients in whom pulmonary complications (abscess/pneumonia) were also more prevalent. In our patients, quality of life (daily activities, pain, emotional problems, and nervousness) and functional outcome scores were lower than the national average. In addition the type of ER did not affect functional outcome or quality of life.[8]

We concluded that most patients are symptomatic after ER, following esophagectomy due to esophageal cancer, when evaluated through a self assessment questionnaire. Dysphagia, heartburn, reflux, regurgitation, gastric dumping and abdominal cramps are common. Most symptoms are mild to moderate and less than half of these patients require medication and dilatation therapy to tolerate symptoms. Last but not least, quality of life and functional outcome after an ER operation is influenced by age, sex, location of anastomosis, and type of ER performed.
